# Gut microbiota and metabolomics in metabolic dysfunction-associated fatty liver disease: interaction, mechanism, and therapeutic value

**DOI:** 10.3389/fcimb.2025.1635638

**Published:** 2025-07-23

**Authors:** Luyu Wang, Hongtao Wang, Jian Wu, Changyi Ji, Ying Wang, Mengmeng Gu, Miaomiao Li, Hongwei Yang

**Affiliations:** ^1^ Anhui Province Key Laboratory of Immunology in Chronic Diseases, Research Center of Laboratory, School of Laboratory, Bengbu Medical University, Bengbu, China; ^2^ Department of Clinical Laboratory, The Affiliated Suzhou Hospital of Nanjing Medical University, Suzhou Municipal Hospital, Gusu School, Nanjing Medical University, Suzhou, Jiangsu, China; ^3^ Suzhou Key Laboratory of Intelligent Critical Illness Biomarkers Translational Reserach, Suzhou, Jiangsu, China; ^4^ Department of Infection Management, The Affiliated Suzhou Hospital of Nanjing Medical University, Suzhou Municipal Hospital, Gusu School, Nanjing Medical University, Suzhou, Jiangsu, China; ^5^ Department of Clinical Laboratory, Suzhou BOE Hospital, Suzhou, Jiangsu, China

**Keywords:** metabolic dysfunction-associated fatty liver disease (MAFLD), gut microbiota, metabolomics, gut-liver axis, precision medicine

## Abstract

The global epidemic of Metabolic dysfunction-associated fatty liver disease (MAFLD) urgently demands breakthroughs in precision medicine strategies. Its pathogenesis centers on the cascade dysregulation of the gut microbiota-metabolite-liver axis: microbial dysbiosis drives hepatic lipid accumulation and fibrosis by suppressing short-chain fatty acid synthesis, activating the TLR4/NF-κB inflammatory pathway, and disrupting bile acid signaling. Metabolomics further reveals characteristic disturbances including free fatty acid accumulation, aberrantly elevated branched-chain amino acids (independently predictive of hepatic steatosis), and mitochondrial dysfunction, providing a molecular basis for disease stratification. The field of precision diagnosis is undergoing transformative innovation—multi-omics integration combined with AI-driven analysis of liver enzymes and metabolic biomarkers enables non-invasive, ultra-high-accuracy staging of fibrosis. Therapeutic strategies are shifting towards personalization: microbial interventions require matching to patient-specific microbial ecology, drug selection necessitates efficacy and safety prediction, and synthetically engineered “artificial microbial ecosystems” represent a cutting-edge direction. Future efforts must establish a “multi-omics profiling–AI-powered dynamic modeling–clinical validation” closed-loop framework to precisely halt MAFLD progression to cirrhosis and hepatocellular carcinoma by deciphering patient-specific mechanisms.

## 1 Introduction

Metabolic dysfunction-associated fatty liver disease (MAFLD), previously termed non-alcoholic fatty liver disease (NAFLD), represents the most prevalent chronic liver disease globally, affecting approximately 32.4% of the population (Riazi et al., 2022). It is closely associated with obesity, insulin resistance, and type 2 diabetes (Hu et al., 2020). International consensus recommends the nomenclature shift to MAFLD to emphasize its underlying metabolic dysregulation (Lazarus et al., 2024). MAFLD progression encompasses hepatic steatosis, inflammation, and fibrosis (Koliaki et al., 2015; Chen and Vitetta, 2020). Recent research highlights the pivotal role of the gut-liver axis: gut dysbiosis, characterized by an elevated Firmicutes/Bacteroidetes ratio (Jasirwan et al., 2021), modulates hepatic inflammation and metabolism through microbial metabolites (Boursier et al., 2016; Aron-Wisnewsky et al., 2020). Specifically, microbiota-derived secondary bile acids regulate lipid metabolism via the FXR signaling pathway (Huang and Kong, 2021), short-chain fatty acids (SCFAs) influence energy balance (Khan et al., 2021), and lipopolysaccharide (LPS) activates the hepatic TLR4 pathway, driving inflammation and fibrosis (Di Vincenzo et al., 2024). Gut barrier dysfunction and subsequent bacterial translocation exacerbate these processes (Martín-Mateos and Albillos, 2021). Diagnostic approaches have undergone significant innovation: while liver biopsy remains the gold standard (Wei et al., 2024), non-invasive strategies have evolved from traditional biomarkers (e.g., TG/HDL-C ratio (Wang et al., 2024), serum Biglycan (Cengiz et al., 2021), and BARD score (Vilar-Gomez and Chalasani, 2018)) towards a new era of multi-omics integration. Nychas et al. (2025) identified nine cross-ethnicity conserved microbial signatures (e.g., enrichment of pathobionts and depletion of protective bacteria) across seven global cohorts (n=1,892), achieving an AUC of 0.95 for distinguishing MAFLD with high inter-ethnic specificity (Nychas et al., 2025). The Xu team pioneered a plasma metabolomics-clinical parameter combined model, demonstrating superior predictive efficacy for severe liver outcomes compared to traditional tools like FIB-4 and NFS (Xu et al., 2025). Therapeutically, probiotics and symbiotic show potential through microbiota modulation (Liu et al., 2020; Carpi et al., 2022; Rong et al., 2023a). However, addressing individual heterogeneity and mechanistic complexity necessitates precision strategies driven by multi-omics approaches.

**Figure 1:**
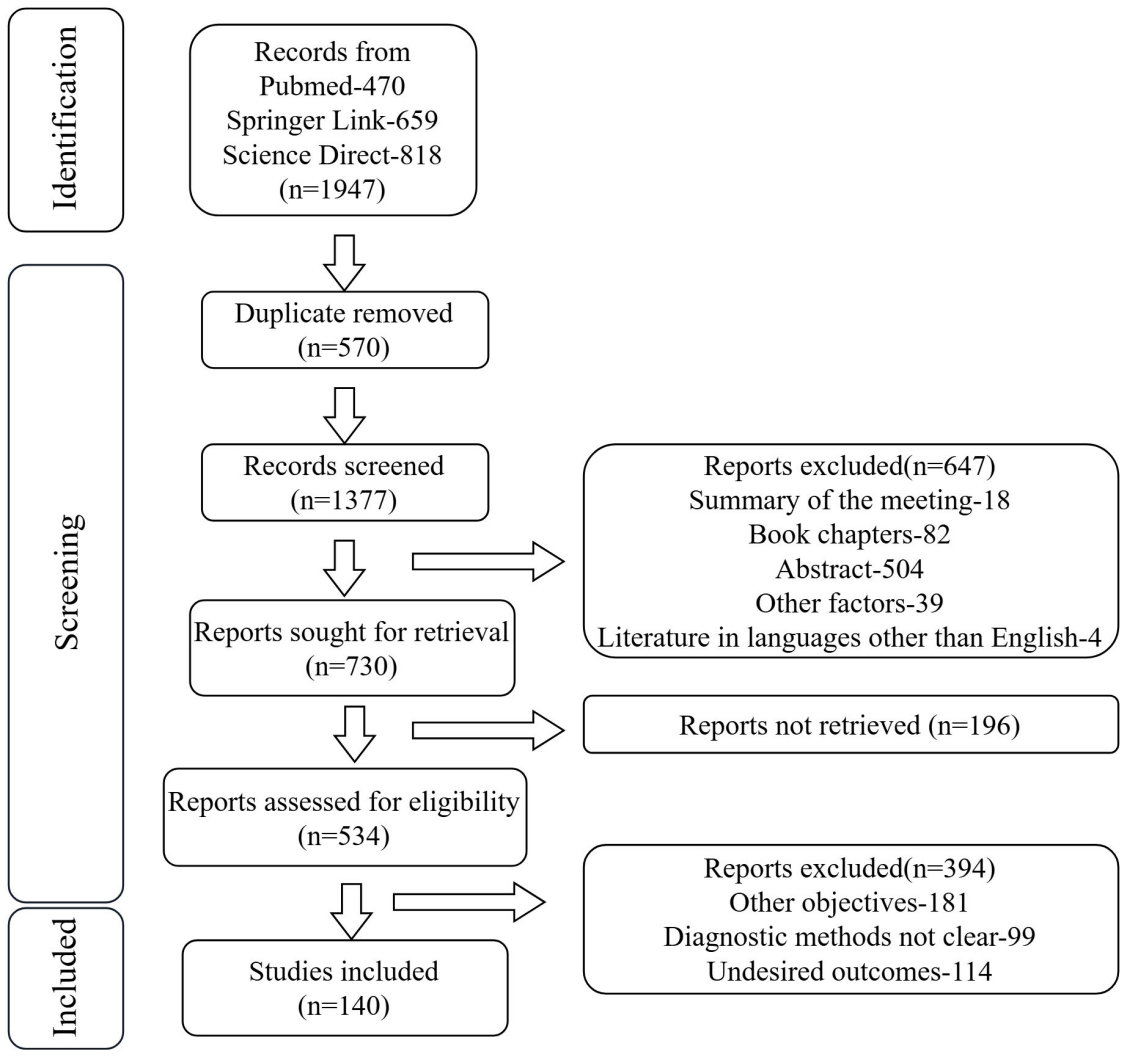
Flowchart of literature selection process in this study.

This study aims to systematically elucidate the role of the gut-microbiota-metabolite-liver axis in MAFLD pathogenesis through integrated multi-omics analysis, providing a theoretical foundation for early diagnosis, risk stratification, and precision intervention strategies. We searched the pubmed, spring link and science direct databases for the past year, and found a total of 1947 articles, including 470 PubMed articles, 659 spring link articles, and 818 science direct articles, and finally we selected 140 relevant articles for research (**Figure 1**).

## 2 Gut microbiota and metabolic dysfunction-associated fatty liver disease

### 2.1 Characteristics of gut microbiota in patients with MAFLD

The development and progression of MAFLD are closely linked to gut dysbiosis. Alterations in the gut microbiota exhibit taxonomic-level specificity and dynamic changes across disease stages (**Table 1**).

**Table 1:** Gut microbiota alterations in MAFLD patients.

Taxonomic level	Biomarker name	Change in MAFLD/MASH	Disease stage association	Function/mechanism	References
Phylum Level	*Bacteroidetes*	↓ Decreased abundance	MAFLD, MASH	Maintains gut barrier integrity; reduction promotes energy absorption & inflammation	(Jasirwan et al., 2021; Jennison and Byrne, 2021; Forlano et al., 2022)
	*Firmicutes*	↑ Increased abundance	MAFLD, MASH	Enhances energy harvest, pro-inflammatory	(Jasirwan et al., 2021; Jennison and Byrne, 2021; Forlano et al., 2022),
	*Proteobacteria*	↑ Significantly increased	MAFLD, Fibrosis	Pro-inflammatory (e.g., endotoxin release)	(Loomba et al., 2019; Forlano et al., 2022)
	*Actinobacteria*	↑Increased abundance	MAFLD	Associated with dysbiosis	(Loomba et al., 2019)
Family Level	*Enterobacteriaceae*	↑Elevated relative abundance	MAFLD, MASH	Pro-inflammatory (LPS biosynthesis)	(Chen and Vitetta, 2020)
	*Rikenellaceae*	↓ Reduced	Early MAFLD	Loss of anti-inflammatory metabolic functions	(Chen and Vitetta, 2020)
	*Ruminococcaceae*	Variable (↓ in adults, ↑ in children)	MAFLD → Fibrosis	Contradictory fibrosis associations; context-dependent compensatory role	(Boursier et al., 2016; Del Chierico et al., 2017; Lee et al., 2020; Oh et al., 2020; Li et al., 2021)
Genus Level	*Escherichia*	↑ Expansion	MAFLD, MASH	Pro-inflammatory, promotes endotoxemia	(Aron-Wisnewsky et al., 2020; Long et al., 2024)
	*Dorea*	↑ Increased	MAFLD	Pro-inflammatory, disrupts gut barrier	(Aron-Wisnewsky et al., 2020)
	*Faecalibacterium*	↓ Significantly reduced	MAFLD, MASH	Reduced butyrate production, impaired anti-inflammatory function	(Aron-Wisnewsky et al., 2020; Long et al., 2024)
	*Coprococcus*	↓ Decreased	MAFLD	Insufficient SCFA production	(Aron-Wisnewsky et al., 2020)
	*Prevotella*	↓ Reduced	MAFLD	Diminished anti-inflammatory metabolite generation	(Aron-Wisnewsky et al., 2020; Long et al., 2024)
	*Eubacterium rectale*	↑ (moderate MAFLD); ↓ (fibrosis)	MAFLD → Fibrosis	Dual role: compensatory adaptation → profibrotic transition	(Chen and Vitetta, 2020)
Functional Features	SCFA synthesis (e.g., butyrate)	↓ Suppressed	MAFLD → MASH	Gut barrier disruption, promotes inflammation	(Boursier et al., 2016)
	LPS biosynthesis	↑ Activated	MASH, Fibrosis	Drives endotoxemia & oxidative stress	(Boursier et al., 2016)
	Tryptophan metabolism	↑ Aberrantly activated	MASH	Promotes pro-inflammatory mediator production	(Boursier et al., 2016)
Diagnostic Markers	16-genus combination	Stage-specific alterations	Advanced Fibrosis	Non-invasive model (e.g., *Ruminococcus* + *Streptococcus*)	(Loomba et al., 2019; Oh et al., 2020)
	Microbial α-diversity	↓ Decreases with hepatic fat accumulation	MAFLD progression	Correlates with disease severity	(Long et al., 2024)

MAFLD, metabolic dysfunction-associated fatty liver disease; MASH, metabolic dysfunction-associated steatohepatitis.

A common hallmark of dysbiosis is an increased abundance of *Proteobacteria* and *Actinobacteria* phyla, along with an elevated *Firmicutes*/*Bacteroidetes* ratio (Loomba et al., 2019). At the phylum level, MAFLD patients typically show reduced abundance of *Bacteroidetes* and increased abundance of *Firmicutes* and *Proteobacteria* (Forlano et al., 2022), forming a characteristic “Firmicutes/Bacteroidetes imbalance.” In healthy individuals, *Firmicutes* and *Bacteroidetes* dominate, while *Proteobacteria* and others are relatively scarce (Jennison and Byrne, 2021). Disruption of this homeostasis may drive MAFLD progression by promoting energy harvest and inflammatory responses. At the family level, MAFLD patients exhibit an increased relative abundance of *Enterobacteriaceae* and a decrease in *Rikenellaceae* and *Ruminococcaceae*, which possess anti-inflammatory metabolic functions (Chen and Vitetta, 2020). Notably, *Ruminococcaceae* abundance shows a positive association with significant fibrosis, suggesting its dynamic changes correlate with disease severity (Boursier et al., 2016). However, investigations into *Ruminococcaceae* abundance in MAFLD/MASH patients reveal inconsistent trends across populations. Del Chierico et al. (2017) observed a significant increase in *Ruminococcaceae* in children/adolescents with MAFLD or MASH compared to healthy controls. Conversely, a meta-analysis by Li et al. (2021) involving 1,265 subjects (including 577 MAFLD patients from 8 countries) found reduced *Ruminococcaceae* in MAFLD patients. Lee et al. (2020) further highlighted this discrepancy, reporting a negative association between *Ruminococcaceae* abundance and significant fibrosis in non-obese patients—a finding contradictory to Boursier et al (Boursier et al., 2016). These collective data indicate that *Ruminococcaceae* abundance varies significantly depending on patient cohorts and metabolic subgroups. Furthermore, alterations at the genus level are more complex: pro-inflammatory genera such as *Escherichia* and *Dorea* expand, while butyrate-producing genera like *Faecalibacterium*, *Coprococcus*, and *Prevotella* are significantly reduced (Aron-Wisnewsky et al., 2020). The abundance change of *Eubacterium rectale* is particularly unique—it increases in moderate-to-severe MAFLD but decreases sharply when fibrosis develops, suggesting a dual role in compensatory adaptation and profibrotic processes across different pathological stages (Chen and Vitetta, 2020).

As the disease progresses to metabolic dysfunction-associated steatohepatitis (MASH) and fibrosis, functional remodeling of the microbiota intensifies. Metagenomic analysis reveals abnormal activation of tryptophan/phenylalanine metabolism and lipopolysaccharide (LPS) biosynthesis pathways in MASH-associated microbiota, while pathways for cellulose degradation and short-chain fatty acid (SCFA) synthesis (e.g., butyrate) are suppressed (Boursier et al., 2016). This metabolic shift amplifies endotoxemia and oxidative stress via the gut-liver axis, further worsening insulin resistance. Non-invasive diagnostic techniques based on microbial signatures are rapidly advancing; for instance, a 16-genus marker model including *Ruminococcus* and *Streptococcus* significantly improves diagnostic accuracy for advanced fibrosis (Loomba et al., 2019; Oh et al., 2020). Methodologically, targeted 16S rRNA sequencing is commonly used for bacterial community analysis, while 18S rRNA or internal transcribed spacer (ITS) sequencing can profile fungal communities; metagenomic sequencing (mNGS) and probe-capture techniques enhance the detection of low-abundance species. Studies indicate that gut microbial α-diversity in MAFLD patients decreases with increasing hepatic fat accumulation, and meta-analyses reveal a core dysbiotic signature characterized by increased *Escherichia* and *Prevotella*, alongside decreased *Faecalibacterium* and *Ruminococcaceae* (Long et al., 2024). These findings suggest that hierarchical disruptions in microbial composition and function are not only biomarkers for MAFLD but also key pathological drivers of disease progression.

### 2.2 Pathological mechanisms of gut microbiota in patients with MAFLD

**Figure 2:**
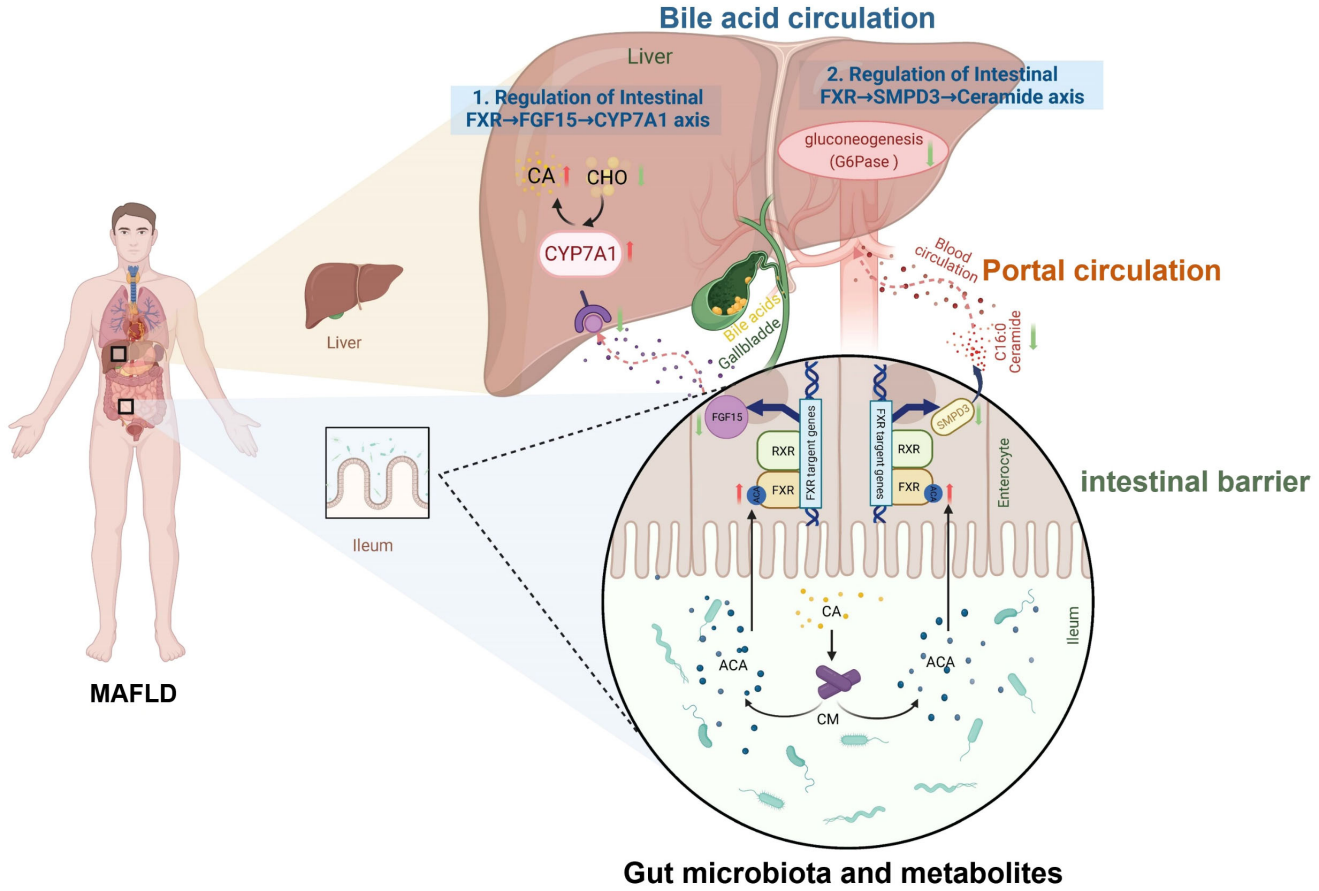
Mechanisms of the gut-liver axis and bile acid metabolism in MAFLD. Bile acids (e.g., CA, cholic acid) derived from hepatic cholesterol (CHO) metabolism are synthesized via CYP7A1 (cholesterol 7α-hydroxylase). They enter intestinal circulation and activate the Farnesoid X Receptor (FXR), inducing fibroblast growth factor 15/19 (FGF15/19). This suppresses hepatic CYP7A1 via portal feedback, completing the enterohepatic loop. The axis interacts with gut microbiota metabolites and influences intestinal barrier integrity. Dysregulation of this pathway (highlighted in MAFLD-condition) links gut-liver crosstalk, microbial metabolites, and barrier dysfunction to disease progression.

MAFLD is characterized by excessive hepatic triglyceride accumulation (Rong et al., 2023b). Its pathological progression is closely linked to gut-liver axis dysfunction driven by gut dysbiosis. The gut-liver axis forms a bidirectional regulatory network via the portal circulation, bile acid metabolism, and immune signaling (Albillos et al., 2020; Wu et al., 2022; Xiang et al., 2023; Siddiqui et al., 2025). Dysregulation of microbial metabolites, gut barrier impairment, and bile acid signaling imbalance constitute three core mechanisms driving hepatic lipid metabolism abnormalities, inflammation activation, and fibrosis (Blesl and Stadlbauer, 2021) (**Figure 2**).

#### 2.2.1 Dysregulation of microbial metabolites

Gut microbiota ferment dietary fibers to generate short-chain fatty acids (SCFAs), uch as butyrate and propionate. These activate the hepatocyte GPR43 receptor, inhibit histone deacetylases (HDACs), upregulate PPARα to promote fatty acid oxidation, and enhance leptin signaling to suppress SREBP-1 and cholesterol synthesis gene expression, thereby reducing hepatic lipid accumulation (Li et al., 2024). Tryptophan metabolites derived from gut microbiota (e.g., indole derivatives) delay hepatic stellate cell (HSC) activation by activating the aryl hydrocarbon receptor (AhR) (Venkatesh et al., 2014). Additionally, microbiota convert primary bile acids to secondary bile acids via 7α-dehydroxylation, activating the farnesoid X receptor (FXR) and TGR5 receptor to regulate lipid metabolism (Huang and Kong, 2021). However, MAFLD patients often exhibit downregulated FXR expression (Long et al., 2024) and reduced secondary/primary bile acid ratios (Xie et al., 2022), weakening negative feedback on lipid synthesis and exacerbating steatosis (Zhang et al., 2006).

#### 2.2.2 Gut barrier impairment

Reduced expression of the tight junction protein ZO-1 facilitates translocation of lipopolysaccharide (LPS) and CpG DNA (Giorgio et al., 2014). LPS activates the TLR4 receptor on Kupffer cells, triggering the release of pro-inflammatory factors (e.g., NF-κB, JNK/AP1) (Stephens and von der Weid, 2020) and disrupting intestinal epithelial junctions, forming a “gut leak-LPS leakage-inflammation” vicious cycle (Wu et al., 2019). Translocated CpG DNA induces insulin resistance via hepatocyte TLR9 (Tripathi et al., 2018), while pathobiont-derived toxic metabolites directly damage hepatocytes (Hu et al., 2020). Clinical studies confirm that intestinal permeability positively correlates with hepatic steatosis in MAFLD, and blood microbial translocation markers are elevated (Cui et al., 2019; De Munck et al., 2020).

#### 2.2.3 Bile acid signaling imbalance

Chenodeoxycholic acid (CDCA) activates TGR5 to promote HSC collagen synthesis (Saga et al., 2018), while deoxycholic acid (DCA) induces hepatocyte apoptosis via the NF-κB/miR-21/PDCD4 pathway (Rodrigues et al., 2015). Dysbiosis-induced reduction of secondary bile acids and increased DCA/CDCA ratio (Xie et al., 2022) not only impair FXR-mediated suppression of lipogenesis but also exacerbate inflammation by disrupting gut immune homeostasis (Cai et al., 2022). TLR signaling plays a central role: TLR4 amplifies inflammation through MyD88-dependent (activating NF-κB, JNK/AP1) and TRIF-dependent pathways (Giorgio et al., 2014; Stephens and von der Weid, 2020). Hepatic lipid accumulation enhances TLR4 sensitivity to LPS, creating a “lipid accumulation → inflammation → metabolic dysregulation” vicious cycle (Huang et al., 2012). TLR9 regulates HSC function by recognizing CpG DNA, driving collagen deposition during chronic injury (Saga et al., 2018; Cui et al., 2019; De Munck et al., 2020). Animal studies show TLR4 knockout alleviates liver injury (Hu et al., 2020), and clinical research confirms TLR4 mRNA levels in MAFLD liver tissue correlate with inflammation/fibrosis severity (Sharifnia et al., 2015).

Collectively, these findings demonstrate that MAFLD pathogenesis involves a network of microbiota-derived metabolites, gut barrier dysfunction, and TLR-mediated immune responses. Metabolic imbalance and amplified inflammation create a positive feedback loop that accelerates disease progression, while the dual roles of TLR signaling and bile acid dysregulation further exacerbate hepatic fibrosis. Targeting gut barrier repair, modulating microbiota composition to restore protective metabolites, and precision intervention in key TLR signaling pathways represent promising strategies for treating MAFLD pathology.

### 2.3 Application of gut microbiota in the treatment of metabolic dysfunction-associated fatty liver disease

Recent studies have revealed the multifaceted mechanisms by which gut microbiota and their metabolic regulation contribute to treating MAFLD. *Lactobacillus* and *Bifidobacterium* significantly reduce serum cholesterol levels by modulating host metabolic pathways, likely through inhibiting intestinal cholesterol absorption and promoting bile acid excretion (Wu and Chiou, 2021). Further research indicates functional differentiation in liver farnesoid X receptor (FXR) subtypes during lipid metabolism regulation. FXRα2 exhibits stronger triglyceride (TG)-inhibiting capacity than FXRα1 via specific binding to DNA motifs, suggesting that targeted selective activation of FXR subtypes may become a novel therapeutic strategy for MAFLD (Ramos Pittol et al., 2020).

In probiotic combination interventions, a mixture of six probiotics (including *Lactobacillus* and *Bifidobacterium*) significantly increased the abundance of beneficial bacteria such as *Agathobaculum*, *Blautia*, and *Ruminococcus* in the gut while reducing hepatic free fatty acids (FFA) and body mass index (BMI), demonstrating the synergistic role of microbiota in ameliorating metabolic disorders (Fang et al., 2022). Exercise intervention reshapes gut microbiota structure, such as reducing *Parabacteroides* and *Flavobacterium*, to enhance hepatic fatty acid oxidation capacity. Independent of weight loss, exercise suppresses the NF-κB inflammatory pathway, thereby reducing intrahepatic lipid accumulation (Ortiz-Alvarez et al., 2020).

For targeted microbial therapies, specific probiotic strains like *Lactobacillus rhamnosus* GG (LGG) inhibit intestinal NF-κB signaling to reduce systemic inflammation, while their metabolites activate the FGF21-adiponectin axis to promote lipid metabolism (Liu et al., 2020) and stimulate butyrate-producing bacteria proliferation to repair the gut barrier (Zhao et al., 2019). *Lactococcus lactis* subsp. *cremoris* outperforms LGG in ameliorating high-fat-induced metabolic dysregulation, evidenced by reduced serum cholesterol, attenuated hepatic steatosis, and restored glucose tolerance (Naudin et al., 2020). The multi-strain probiotic VSL#3 alleviates liver inflammation by suppressing the NF-κB pathway and downregulating key lipogenesis genes (SREBP-1c and *FAS*) (Jena et al., 2020). Prebiotics and synbiotics not only enhance fatty acid β-oxidation by upregulating PPAR-α/CPT-1 but also inhibit colonization of pro-inflammatory bacteria such as *Enterobacteriaceae*, thereby improving insulin resistance and liver injury (Alves et al., 2017). These findings highlight the potential of precision intervention strategies based on microbiota-host interactions in MAFLD management.

Current clinical research on fecal microbiota transplantation (FMT) for MAFLD remains exploratory. Three key trials reveal its potential and limitations: Craven et al. (2020) found that allogeneic FMT significantly improved intestinal permeability in MAFLD patients, though without improving HOMA-IR or MRI-PDFF. Witjes et al. (2020) demonstrated that FMT from healthy donors upregulated hepatic *ARHGAP18* (a cytoskeleton regulator) and serine dehydratase (*SDS*) expression in patients with MASH while reducing serum GGT and ALT. Xue et al. (2022) reported decreased CAP values post-FMT alongside proliferation of butyrate-producing bacteria, activation of the FXR/TGR5 axis, and inhibition of fatty acid synthase (*FASN*). These results suggest FMT may mitigate liver injury by repairing the gut barrier, regulating host gene expression, and modulating metabolic pathways. However, heterogeneous efficacy, long-term safety concerns, and insufficient mechanistic validation (Qiu et al., 2024) require resolution through standardized donor screening and optimized trial designs.

Emerging gut-liver axis strategies indicate that symbiotic supplementation enriches butyrate-producing microbiota, elevates short-chain fatty acid (SCFA) levels, improves insulin resistance, inhibits hepatic lipogenic enzymes, and alleviates inflammation/oxidative stress via FXR/TGR5 signaling (Eslamparast et al., 2014). This underscores the potential of microbiota modulation to reshape gut-liver metabolic crosstalk, offering a microbe-centric paradigm for MAFLD.

The field of microbiota-targeted therapy is evolving from single-strain supplementation toward systematic ecological modulation. Future advances demand prioritizing functional gene clusters over individual species, establishing real-time monitoring of dynamic microbiota-host interactions, and leveraging synthetic biology to design therapeutic artificial microbial ecosystems. We prioritize butyrate synthesis (e.g., but/buk gene clusters) (Kalkan et al., 2025)and bile acid metabolism (e.g., bai/bsh genes) (Li et al., 2023) as core therapeutic targets due to their direct regulation of intestinal barrier integrity, host immunity, and metabolic homeostasis; concurrently, short-chain fatty acid transporters and antimicrobial peptide synthesis gene clusters will be incorporated to enhance microbial colonization resistance. Clinical efficacy will be evaluated via a multidimensional strategy: metagenomic tracking of functional gene abundance, metabolomic quantification (GC-MS/LC-MS) of butyrate and bile acid metabolites, host-response analysis of serum inflammatory markers and intestinal barrier indicators, and systematic correlation with clinical symptom scores to validate therapeutic mechanisms and translational potential. Ultimately, by redefining the microbiome as a programmable biological network, precise strategies for MAFLD prevention and treatment can be achieved.

## 3 Metabolomics and metabolic dysfunction-associated fatty liver disease

### 3.1 Metabolomic signatures in MAFLD patients

Metabolomic studies reveal significant metabolic dysregulations in patients with MAFLD, involving multiple pathways such as lipid, amino acid, bile acid, and energy metabolism. These alterations are closely linked to disease progression. MAFLD patients commonly exhibit hepatic lipid deposition, characterized by elevated free fatty acid (FFA) levels (Guo et al., 2022), increased triglyceride/high-density lipoprotein cholesterol (TG/HDL-C) ratio (a non-invasive diagnostic marker) (Fan et al., 2019), and phospholipid imbalance (e.g., decreased phosphatidylcholine/phosphatidylethanolamine (PC/PE) ratio) (Peng et al., 2021). Impaired hepatic mitochondrial β-oxidation leads to long-chain fatty acid accumulation, exacerbating lipotoxicity (Koliaki et al., 2015).

Dysregulated branched-chain amino acid (BCAA) metabolism is a hallmark feature, with elevated blood levels of BCAAs (e.g., leucine, isoleucine) and their metabolites correlating with insulin resistance and hepatic steatosis (Lo et al., 2022). Concurrently, increased aromatic amino acids (e.g., phenylalanine, tyrosine) and glutamate may promote inflammation and fibrosis via mTOR pathway activation (Samuel et al., 2004). Gut microbiota dysbiosis (e.g., elevated *Firmicutes*/*Bacteroidetes* ratio) (Jasirwan et al., 2021) (Boursier et al., 2016) disrupts the gut-liver axis through bile acid metabolism, resulting in increased secondary bile acids (e.g., deoxycholic acid) and reduced primary bile acids (e.g., taurocholic acid). This impairs farnesoid X receptor (FXR) signaling, worsening lipid dysregulation and inflammation (Wang et al., 2025).

While mitochondrial adaptive responses persist in simple steatosis (e.g., compensatory enhanced fatty acid oxidation), progression to MASH reduces oxidative phosphorylation efficiency. Accumulation of tricarboxylic acid (TCA) cycle intermediates (e.g., citrate, succinate) and elevated reactive oxygen species (ROS) production drive cellular damage and fibrosis (Koliaki et al., 2015).

Metabolomics has identified multiple potential biomarkers (**Table 2**), including serum BCAAs, 2-aminoadipic acid (2-AAA), and specific lipid profiles (e.g., Lys phosphatidylcholines), which correlate significantly with hepatic fat content, inflammation, and fibrosis severity (Di Mauro et al., 2021). Integrating these with machine learning models (e.g., laboratory parameter-based MAFLD screening) (Yip et al., 2017) or traditional scoring systems (e.g., BARD score) (Rigor et al., 2022) enhances diagnostic and staging accuracy.

**Table 2:** Metabolic dysfunction-associated biomarkers.

Indicator name	Category	Application	Advantage	Key parameters/features	Related study	Reference
TG/HDL-C ratio	Blood biochemical marker	Predict MAFLD	Simple, effective surrogate	Ratio calculation	Fan et al.	(Fan et al., 2019)
Serum BGN (Biglycan)	Serum marker	Diagnose MASH and significant fibrosis	Non-invasive, novel biomarker	High specificity	Cengiz et al.	(Cengiz et al., 2021)
Arachidonic acid oxidation products	Metabolic marker	Diagnose MASH	High specificity, reflects oxidative stress	Multi-metabolite panel	Di Mauro et al.	(Di Mauro et al., 2021)
Hepascore (GGT, HA, α2m combination)	Blood biochemical composite	Diagnose advanced fibrosis (F3-F4)	High diagnostic performance	Balanced sensitivity/specificity	Boursier et al.	(Boursier et al., 2016)
miRNA-122 & miRNA-34a	Circulating microRNA	Differentiate MAFLD patients from controls	High AUC (0.93-0.96)	Non-invasive, high accuracy	Hochreuter et al.	(Hochreuter et al., 2022)
MAFLD Ridge Score	Machine learning model	Exclude MAFLD (epidemiological studies)	Comparable to existing scores	Laboratory-parameter based	Yip et al.	(Yip et al., 2017)
HSI (Hepatic Steatosis Index)	Clinical scoring system	Screen MAFLD	Simple, effective for steatosis grading	BMI + ALT + gender	Di Mauro et al.	(Di Mauro et al., 2021)
BARD Score	Clinical scoring system	Diagnose advanced fibrosis	Avoids biopsy, highly applicable	BMI + AST/ALT + diabetes status	Vilar-Gomez et al.	(Vilar-Gomez and Chalasani, 2018)
ALT/AST ratio, FIB-4, MAFLD Fibrosis Score	Non-invasive scoring systems	Exclude advanced fibrosis	High reliability, simplified assessment	Multi-parameter evaluation	Vilar-Gomez et al.	(Vilar-Gomez and Chalasani, 2018)

MAFLD, metabolic dysfunction-associated fatty liver disease; MASH, metabolic dysfunction-associated steatohepatitis; ALT, alanine aminotransferase; AST, glutamic oxaloacetic transaminase; BMI, body mass index; TG/HDL-C, triglyceride/high-density lipoprotein cholesterol.

Thus, metabolomics not only provides molecular insights into MAFLD pathogenesis but also enables novel approaches for non-invasive diagnosis, disease subtyping, and targeted therapies (e.g., FXR agonists, gut microbiota modulation) (Xu et al., 2025). Future research should integrate multi-omics data to precisely delineate metabolic network dynamics in MAFLD progression.

### 3.2 Mechanisms of host metabolism in metabolic dysfunction-associated fatty liver disease

**Figure 3:**
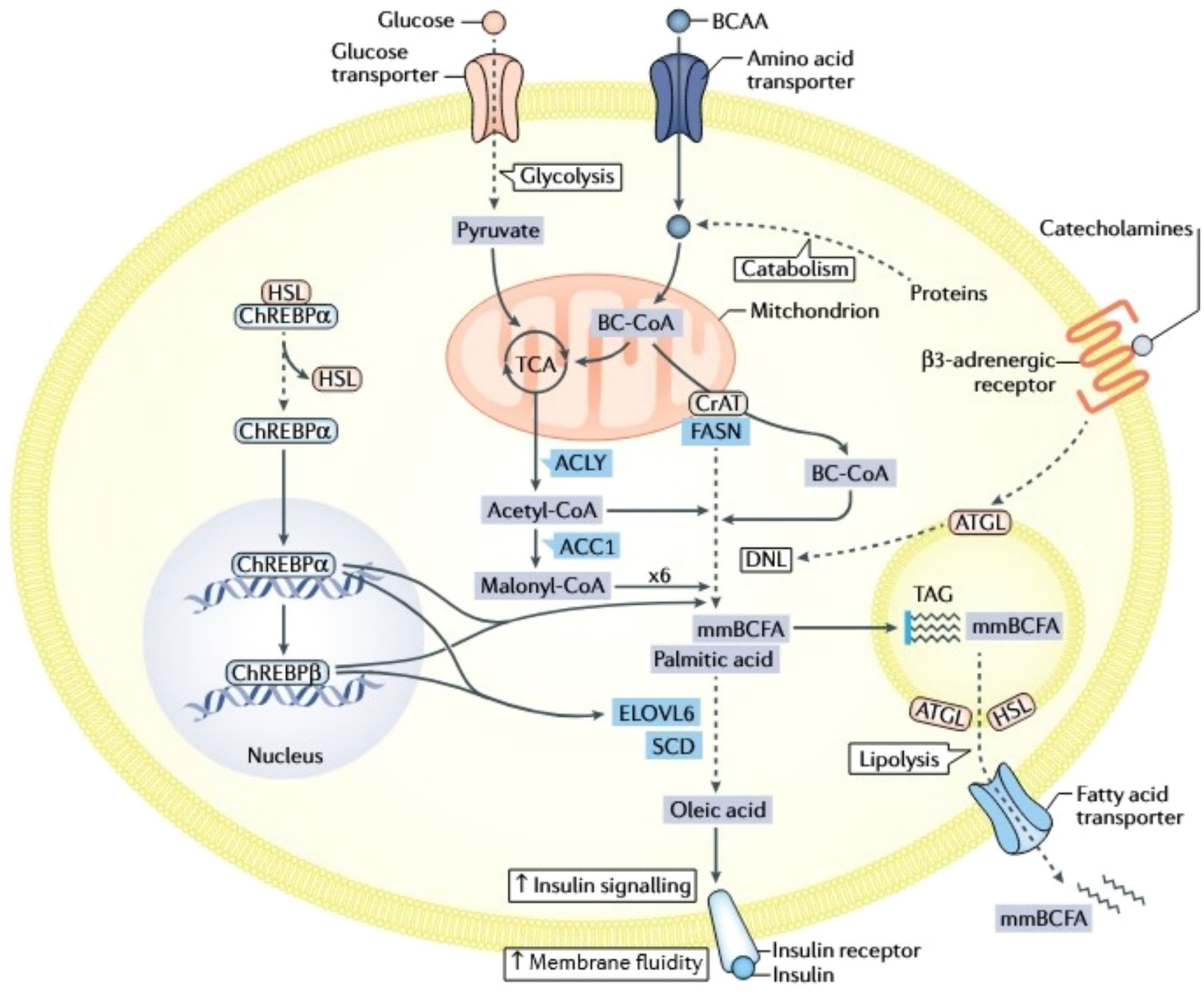
Integrated metabolic network of glucose transport, lipid synthesis, and insulin signaling. Cellular glucose uptake, facilitated by Glucose Transporters (GLUT), fuels glycolysis to generate pyruvate and also regulates the transport of Branched-Chain Amino Acids (BCAAs), creating a fundamental link between carbohydrate and amino acid metabolism. Glucose-derived metabolites, notably acetyl-CoA, activate the Carbohydrate-Responsive Element-Binding Protein (ChREBP), which drives *de novo* lipogenesis by upregulating key enzymes: Acetyl-CoA Carboxylase (ACC) and Fatty Acid Synthase (FAS) for palmitic acid synthesis, and Diacylglycerol Acyltransferase (DGAT) for Triacylglycerol (TAG) assembly. Concurrently, lipid metabolism involves the release of free fatty acids via lipolysis, their cellular transport via specific transporters, and their utilization in pathways like β-oxidation or modulation of membrane fluidity. The Insulin Signaling Pathway is central to coordinating this metabolic network; insulin receptor activation promotes glucose uptake and anabolic processes, but impaired signaling disrupts critical functions including membrane fluidity, receptor efficacy, and overall metabolic homeostasis. This network features significant cross-talk, particularly where BCAA metabolism intersects with glucose flux and lipid synthesis pathways. Additionally, catecholamines (e.g., adrenaline) influence energy balance by activating β3-adrenergic receptors, which modulate lipolysis and energy expenditure, further integrating hormonal control with core metabolic processes.

Metabolomics systematically analyzes dynamic changes in endogenous metabolites to elucidate the pathological mechanisms of MAFLD. This metabolic disorder, characterized by hepatic lipid accumulation, involves complex pathogenesis encompassing dysregulated lipid, amino acid, and carbohydrate metabolism. Metabolomics thus provides novel insights into these disturbances (**Figure 3**).

The core etiology of MAFLD stems from disrupted hepatic lipid metabolism, primarily characterized by excessive triglyceride (TG) accumulation. This steatosis develops when lipid metabolic capacity becomes overwhelmed due to an imbalance between lipogenesis and degradation pathways, resulting in abnormal lipid deposition within hepatocytes (Chen et al., 2019). Excessive hepatic free fatty acid (FFA) accumulation serves as a key driver of this process, originating from three interconnected sources: adipose tissue lipolysis increasing circulating FFA levels (closely linked to insulin resistance [IR] and hepatic inflammation) (Griffin et al., 1999); hyperactive *de novo* lipogenesis (DNL) where skeletal muscle IR-induced hyperglycemia and hyperinsulinemia activate transcription factors ChREBP and SREBP1c, upregulating lipogenic enzymes that convert excess glucose into FFA (Samuel and Shulman, 2018); and dietary lipids entering the liver through bile acid receptor-mediated absorption (e.g., via FXR), further exacerbating FFA burden (Cheng et al., 2024).FFA overaccumulation not only impairs mitochondrial β-oxidation (e.g., through CPT1 downregulation (Serviddio et al., 2011)) but also promotes oxidative stress and hepatic fibrosis (Ramanathan et al., 2022). Beyond FFA dysregulation, other lipid abnormalities contribute to MAFLD progression. An imbalanced phosphatidylcholine-to-phosphatidylethanolamine (PC/PE) ratio disrupts membrane integrity, with decreased ratios distinguishing simple steatosis from MASH and liver injury (Peng et al., 2021). Mitochondrial adaptive responses (e.g., PPARα and CPT1 upregulation) may initially enhance fatty acid oxidation, but these compensatory mechanisms progressively fail amid evolving IR and hormonal changes like leptin dysregulation (Begriche et al., 2013).

Amino acid metabolic disturbances critically influence MAFLD pathogenesis, particularly the branched-chain amino acid (BCAA) and aromatic amino acid (AAA) imbalance. BCAA dysregulation activates the mammalian target of rapamycin (mTOR) pathway, exacerbating IR and hepatocyte steatosis (Lo et al., 2022). BCAT2 knockout models demonstrate that BCAA accumulation induces energy metabolism disorders while paradoxically conferring obesity resistance, revealing its dual metabolic roles (Ananieva et al., 2017). Clinical studies confirm significant positive correlations between serum BCAA levels and intrahepatic triglyceride content (IHTC), ALT, AST, and GGT in MAFLD patients. Critically, the BCAA-IHTC association persists after adjusting for obesity and IR, indicating BCAA’s direct steatogenic role (Ni et al., 2023) (van den Berg et al., 2019). *In vitro* validation shows valine upregulates lipogenic transcription factors (e.g., SREBP-1c), promoting lipid synthesis while inhibiting fatty acid oxidation to increase hepatocellular TG (Ni et al., 2023). Notably, obese MAFLD patients exhibit higher BCAA elevations than non-obese counterparts, with valine and isoleucine accumulation directly correlating with hepatic fat content (Lischka et al., 2020). High BCAA intake also correlates with liver injury severity in obese MAFLD patients, highlighting diet-metabolism interactions (Galarregui et al., 2021).

AAA metabolic abnormalities associate closely with hepatic inflammation and fibrosis in MASH, potentially through pro-inflammatory pathway activation (e.g., NF-κB) (Kalhan et al., 2011). Excessive glutamine breakdown causes ammonia accumulation, impairing hepatocyte function. Recent evidence reveals ammonia promotes SREBP-1 maturation and lipogenesis by activating SCAP/Insig complex dissociation, elucidating its molecular role in MAFLD/MASH (Cheng et al., 2022). This process interfaces with gut microbiota metabolism, as elevated serum BCAA correlates with dysbiosis and IR (Pedersen et al., 2016), positioning the “gut microbiota-amino acid-liver” axis as central to MAFLD. Collectively, amino acid dysregulation orchestrates MAFLD pathology by modulating lipid synthesis, inflammation, and energy metabolism.

Carbohydrate metabolism dysregulation represents another hallmark of MAFLD, manifesting through coordinated glycolysis and gluconeogenesis dysfunction. Elevated blood lactate and pyruvate in MAFLD patients indicate disordered hepatic glucose metabolism and mitochondrial impairment (Koliaki et al., 2015). Dietary patterns critically drive this imbalance: high-glycemic-index (GI) diets induce postprandial hyperglycemia, stimulating hepatic DNL and lipid accumulation (Parker and Kim, 2019). Excessive monosaccharide/disaccharide intake (e.g., fructose, sucrose, glucose) promotes MAFLD progression primarily through ChREBP activation (Katz et al., 2021). As a central lipogenic transcription factor, ChREBP directly binds promoters of DNL enzymes like fatty acid synthase (FASN) and acetyl-CoA carboxylase (ACC) (Ortega-Prieto and Postic, 2019). High-carbohydrate diets enhance ChREBP nuclear translocation and synergism with SREBP-1c, driving postprandial metabolic reprogramming (Linden et al., 2018). In 30%-sucrose-fed mouse models, aberrant ChREBP activation increases hepatic lipid droplets and inflammatory signaling—phenotypes partially reversed by ChREBP inhibition (Daniel et al., 2021). ChREBP also mediates fructose-induced gluconeogenesis dysregulation via insulin-independent mechanisms, indicating its unique role in metabolic compensation (Kim et al., 2016).

Investigation of these pathological mechanisms reveals that MAFLD’s metabolic disturbances involve multidimensional crosstalk. Insulin resistance acts as the central hub, coordinating synergistic dysregulation across lipid, amino acid, and carbohydrate metabolism to promote concurrent hepatocellular injury, inflammation, and fibrogenesis—ultimately driving progression from steatosis to MASH.

### 3.3 Therapeutic applications of metabolomics in metabolic dysfunction-associated fatty liver disease

Metabolomics research provides a systemic perspective for elucidating the pathogenesis of MAFLD and developing clinical interventions, driving a paradigm shift from single-pathway targeting toward systemic network modulation. At the foundational intervention level, scientific dietary management remains central: low-fat, high-fiber diets alleviate intrahepatic lipid deposition by optimizing metabolic profiles, while ω-3 polyunsaturated fatty acid (EPA/DHA)-rich regimens significantly reduce hepatic triglycerides, enhance insulin sensitivity, and suppress inflammation (Scorletti and Byrne, 2018). The Mediterranean diet, rich in olive oil, nuts, and deep-sea fish, demonstrates efficacy by modulating lipid metabolism, reducing liver enzymes such as ALT and AST, and attenuating hepatic inflammation (Gantenbein and Kanaka-Gantenbein, 2021). More recently, the ketogenic diet—characterized by very low carbohydrate and high fat intake—has been shown to improve MAFLD through enhanced lipid oxidation and reduced hepatic lipogenesis (Watanabe et al., 2020). Exercise functions as a synergistic metabolic modulator, improving glucose-lipid metabolism and reducing intrahepatic fat content (Vanweert et al., 2021), with combined resistance and aerobic training yielding superior outcomes in both non-obese and obese MAFLD patients (Zhang et al., 2022).

Pharmacological strategies for MAFLD and metabolic dysfunction-associated steatohepatitis (MASH) exhibit multi-tiered advances. Classic insulin sensitizers like metformin improve underlying metabolic abnormalities by regulating glucose-lipid metabolism, though evidence for histological improvement such as fibrosis reversal in MASH remains limited (Ruan et al., 2023). Conversely, the PPARγ agonist pioglitazone significantly reduces hepatic steatosis, lobular inflammation, and hepatocyte ballooning in non-diabetic MASH patients while delaying diabetes progression (Cusi et al., 2016). Among emerging targeted agents, the bile acid-fatty acid conjugate Aramchol inhibits SCD1 to reduce lipid synthesis, with its Phase III ARMOR trial (NCT04104321) for F2-F3 fibrosis MASH patients currently evaluating efficacy (Alkhouri et al., 2021). The FXR agonist Obet cholic acid (OCA), a selective bile acid modulator, significantly improved MASH-related fibrosis (≥1-stage improvement without worsening) at 25 mg/day in Phase III trials, though approximately 20% of patients discontinued treatment due to pruritus (Chiang and Ferrell, 2022). Notably, the GLP-1 receptor agonist semaglutide demonstrated substantial advantages in a Phase II trial where 0.4 mg daily treatment for 72 weeks achieved histological resolution without worsening fibrosis in 320 MASH patients, positioning it as the most promising metabolic-regulating therapy to date (Zhang et al., 2025). For severely obese patients, foregut bariatric surgery is recommended by international guidelines as an effective intervention (European Association for the Study of the Liver (EASL) et al., 2016), significantly improving BMI, fibrosis scores, and histological features (Nickel et al., 2018), while statins serve as adjunctive therapy for dyslipidemia comorbidities but remain contraindicated in decompensated cirrhosis (Chalasani et al., 2018).

In summary, the current therapeutic framework integrates foundational lifestyle interventions, precision medications targeting the gut-liver axis such as OCA and semaglutide, and surgical approaches, highlighting the necessity for metabolomics-driven individualized treatment selection. Semaglutide demonstrates superior histological resolution and safety profiles, whereas OCA improves fibrosis but faces limitations due to side effects. Future exploration of combination strategies—particularly GLP-1 and FXR agonist synergism—is warranted to cooperatively regulate multiple pathological pathways and optimize therapeutic outcomes.

## 4 Summary and outlook

Despite progress in elucidating gut-liver axis mechanisms and metabolomic features of MAFLD, significant challenges persist. Heterogeneity in microbiota research constitutes a primary obstacle, with current conclusions largely derived from small-sample cross-sectional studies vulnerable to technical variations like sensitivity differences between 16S rRNA and metagenomic sequencing, and population-specific metabolic contexts such as obese versus non-obese subtypes. This compromises reproducibility and generalizability, exemplified by inconsistent Ruminococcaceae abundance patterns—elevated in pediatric MAFLD yet reduced in adult meta-analyses, with paradoxical fibrosis correlations—highlighting context-dependent microbiota-host interactions.

Metabolomic platform variability similarly hinders translation due to unstandardized detection techniques and analytical pipelines, while cross-regulatory metabolic pathways diminish single-metabolite biomarker specificity. Although machine learning models integrating lipid profiles and amino acid signatures improve diagnostics, clinical adoption remains limited by technical discrepancies and metabolic network dynamism.

The translational gap is particularly pronounced: While probiotics, FXR agonists, and fecal microbiota transplantation demonstrate efficacy in animal models, human trials show marked heterogeneity. Long-term safety and efficacy of emerging therapies require large-scale validation, and lifestyle interventions lack clarity on long-term fibrotic impacts. Bariatric surgery demands precise patient stratification due to strict indications. Limitations in multimodal data and machine learning exacerbate challenges—inconsistent diagnostic data acquisition across centers, limited model generalizability without external validation, and clinician skepticism regarding “black-box” interpretability impede real-world adoption (Meng et al., 2023; Huang et al., 2025). These issues collectively necessitate a paradigm shift toward multi-omics-driven dynamic network intervention.

Future breakthroughs depend on integrating three synergistic strategies: Cross-omics dynamic network deconvolution will establish causal mechanisms linking strain function to host phenotypes, resolving paradoxes like Ruminococcaceae variability. AI-driven precision management systems will enable full-cycle care—ML models like the NAFLD Ridge Score (AUROC=0.88 (Aggarwal and Alkhouri, 2021)) integrating clinical and multi-omics features for dynamic risk stratification; deep learning fusing liver enzymes, radiomics, and cell death markers for high-accuracy fibrosis staging (Okanoue et al., 2021); and SVM algorithms predicting treatment responses to optimize probiotic dosing or FMT donor selection (Lewinska et al., 2021). Finally, adaptive clinical trials will stratify patients by baseline microbial, metabolic, and genetic profiles to validate targeted therapies, incorporating real-time metabolomic monitoring for efficacy assessment. Only by embedding microbiomes and metabolomes within a systems medicine framework can we bridge the gap from mechanistic exploration to clinical precision in NAFLD, ultimately alleviating the global burden of cirrhosis and hepatocellular carcinoma.
